# Recent advances in the relationships between biofilms and microplastics in natural environments

**DOI:** 10.1007/s11274-024-04021-y

**Published:** 2024-05-29

**Authors:** Eva Ventura, Anna Marín, José Gámez-Pérez, Luis Cabedo

**Affiliations:** Polymers and Advanced Materials Group (PIMA), Universitat Jaume I (UJI), Castelló de la Plana, Castellón Spain

**Keywords:** Environmental impact, Microbial interaction, Plastic, Pollution, Seawater, Soil

## Abstract

Plastic pollution in the form of microplastics (MPs), poses a significant threat to natural ecosystems, with detrimental ecological, social, and economic impacts. This review paper aims to provide an overview of the existing research on the interaction between microbial biofilms and MPs in natural environments. The review begins by outlining the sources and types of MPs, emphasizing their widespread presence in marine, freshwater, and terrestrial ecosystems. It then discusses the formation and characteristics of microbial biofilms on MPs surfaces, highlighting their role in altering the physicochemical properties of MPs and facilitating processes such as vertical transport, biodegradation, dispersion of microorganisms, and gene transfer. Different methods used to assess these interactions are discussed, including microbiological and physicochemical characterization. Current gaps and challenges in understanding the complex relationships between biofilms and MPs are identified, highlighting the need for further research to elucidate the mechanisms underlying these complex interactions and to develop effective mitigation strategies. Innovative solutions, including bioremediation techniques and their combination with other strategies, such as nanotechnology, advanced filtration technologies, and public awareness campaigns, are proposed as promising approaches to address the issue of MPs pollution. Overall, this review underscores the urgent need for a multidisciplinary approach to combating MPs pollution, combining scientific research, technological innovation, and public engagement to safeguard the health and integrity of natural ecosystems.

## Introduction

Since 1907 and the invention of the first synthetic plastic, plastics industry has grown to reach a global production of 400.3 Mt and a turnover of more than 400 billion € in revenue in 2022 (Plastics Europe 2023), this entailing substantial waste generation rates. In recent years, the significant increase in the use of disposable plastic products together with the prevalence of the “throw-away” mindset have exacerbated the issue. If mismanaged, plastic waste may be released into the environment, where it accumulates due to its recalcitrant nature. It is currently estimated that less than 20% of this waste is properly managed and that only one third of the plastic produced in Europe is recycled (European Parliament 2023; Lampitt et al. 2023). The oceans are a major destination for plastics waste, with 10 to 20 Mt of plastic entering the seas each year (Thompson et al 2004; Jambeck et al. 2015; Borrelle et al. 2020).Haga clic o pulse aquí para escribir texto. Less research focuses on freshwater ecosystems, but evidence suggests that plastic pollution in these environments is comparable to marine ecosystems (Peng et al. 2017). Terrestrial ecosystems are also affected by plastic pollution, with agricultural practices and wastewater treatment being some of the main sources (Wong et al. 2020).

In these environments, plastic pollution especially in the form of microplastics (MPs), which were first reported in the late 1960s, pose a serious threat with ecological, social, and economic impacts, and is a global threat that could affect human health and biological diversity in the near- to medium-term future (Ryan 2015; Blettler et al. 2018). Several studies have reported interactions between MPs and a wide range of organisms in both marine and terrestrial environments, showing effects ranging from ingestion and other gastrointestinal problems, reduced fitness, systemic immunological responses, occurrence of severe toxicological effects, reduced fecundity and increased mortality, among many others (Cole et al. 2013; Li et al. 2020; Yuan et al. 2023).

This review provides a summary of the existing research concerning the interplay between microbial biofilms and MPs in natural environments, exploring their sources, types and distribution in marine, freshwater and terrestrial ecosystems. It delves into how biofilms on MPs can alter their properties, facilitating processes like vertical transport, biodegradation, microbial dispersion and gene transfer. Different assessment methods are discussed, while identified gaps and challenges underscore the need for further research to understand these complex interactions and develop effective mitigation strategies. The review also proposes innovative solutions such as bioremediation, nanotechnology, advanced filtration, and public awareness initiatives to urgently address MPs pollution, emphasizing the importance of a multidisciplinary approach that integrates scientific research, technological advances, and public engagement to protect the environment.

## Microplastics: sources and types

The term MP refers to small plastic pieces ranging in size from 1 μm to < 5 mm in diameter. The composition of the MPs found in the environment is greatly varied and heterogeneous, differing in sizes, shapes, and chemical composition. MPs can be classified by origin as primary, created for specific applications such as plastic pellets and microbeads; or secondary, resulting from the fragmentation and degradation of larger plastic pieces (Browne et al. 2011).

MPs enter natural environments through different pathways, including inappropriate use and inadequate management protocols. Nonetheless, regular daily human activities also cause great impact. For instance, domestic wastewaters are a notable source of MPs, originating from cosmetics, and personal hygiene and cleaning products. Additionally, fibers from synthetic textiles released through washing machine wastewater and MPs from tires and road markings are significant sources of these pollutants (Napper and Thompson 2016; Järlskog et al. 2022). Other activities associated with the introduction of MPs into aquatic environments include fishing, aquaculture, shipping, and tourist attractions (Ryan et al. 2019; Chen et al. 2021; Belioka and Achilias 2023). MPs pollution can also be the outcome of certain agricultural practices, including the employment of plastic mulches, plastic-coated seeds, and fertilizers (Steinmetz et al. 2016; Katsumi et al. 2021; Wang et al. 2024b). Consequently, agricultural lands can accumulate high concentrations of MPs, becoming a reservoir and an emitting source that can contaminate other natural compartments. Landfills also contribute significantly to the accumulation of MPs in terrestrial environments by housing vast amounts of plastic waste (Hou et al. 2021; Silva et al. 2021).

Interactions between aquatic and on-land activities facilitate the active transfer of MPs between environments. Surface waters can carry plastic fragments to shores and riverbanks, enhancing their deposition onto soils. There, different processes may enhance their movementinto deeper soil layers or their persistence in surface layers (Duan et al. 2021). In both scenarios, MPs can remain in the soil or encounter new water currents, that could transport them to other locations. Especiallyat the surface, natural processes such as soil erosion can be essential for the movement of MP particles.

The abundance and persistence of MPs in natural environments have been studied. Different works focused on marine and freshwater systems have been able to detect MPs pollution at every level that configures these ecosystems, proving plastic pollution as a generalized threat (Table 1). As for terrestrial ecosystems, MPs pollution is significantly higher (up to 20 times, according to some authors) (Horton et al. 2017b; De Souza Machado et al. 2018). Furthermore, agricultural soils would be the ones affected the most, containing between 1.5 and 6.6 million tons of MPs worldwide (Kedzierski et al. 2023).

**Table 1:** Summary of the types of microplastics found in aquatic and terrestrial environments and their abundance

Environment	Location	Type of MP*	Abundance	Reference
Seawater, sediment	Australia	PA, PET	83 particles/L 3400 particles/kg	Ghanadi et al. (2024)
Seawater, sediment	Greece	PE, PP	0.002 particles/L 734 particles/m^2^	Kermenidou et al. (2023)
Coastal aquifers, seawater, sediment	India	PA, PET, PVC	13 particles/L 4400 particles/kg	Srihari et al. (2023)
Seawater	Spain	CA, PE, PP, PS	2.4 particles/L	Rios-Fuster et al. (2022)
River water, sediment	China	PE, PP	0.0004 particles/L 218 particles/kg	He et al. (2024)
River water, sediment	Italy	PAC, PES, PP, PVC,	0.06 particles/L 26.5 particles/kg	Sbarberi et al. (2024)
Lake sediment	India	HDPE, PA, PC, PET, PP, PS	440 particles/kg	Kumar et al. (2024)
River sediment	UK	PP, PES, PAS	66,000–100000 particles/kg	Horton et al. (2017a)
Floodplain soil	Switzerland	NL, PA, PE, PS, PVC, SBR	593 particles/kg	Scheurer and Bigalke (2018)
Agricultural soil	China	EVA, PE, PET	4050 particles/kg	Jia et al. (2024)
Farmland soil	China	EVA, PE, PET, PLA, PP, PS, PTFE, PU, PVC	3056 particles/kg	Wang et al. (2024b)
Urban soil	UK	PE, PP, PS	15,700 particles/kg	Billings et al. (2023)
Agricultural soil	Spain	PP, PVC	2030 particles/kg	van den Berg et al. (2020)
Agricultural soil	Korea	PE, PET, PP	7630 particles/kg	Kim et al. (2021)
Agricultural soil	Germany	PE, PP, PS	0.4 particles/kg	Piehl et al. (2018)

^*^CA cellulose acetate; *EVA* ethylene vinyl acetate; *HDPE* high-density polyethylene; *PA* polyamide, *PAC* polyacrylate; *PAS* polyarylsulphone; *PC* polycarbonate; *PE* polythyelene, *PES* polyester; *PET* polyethylene terephthalate; *PLA* polylactic acid, *PP* polypropylene; *PTFE* polytetrafluoroethylene; *PVC* polyvinyl chloride; *PU* polyurethane; *SBR* styrene butadiene

Polyethylene (PE), polypropylene (PP), polyvinyl chloride (PVC), PS (polystyrene) and polyethylene terephthalate (PET) account for 90% of the total production of plastics, therefore its presence in the environment is considerably high (Andrady et al. 2011). Table 1 provides an overview of the different types of MPs found and their abundance in various natural environments.

## Microbial biofilms on microplastics

Biofilms are complex structures composed of a variety of microorganisms, embedded in a matrix of extracellular polymeric substances. Existence within a biofilm provides numerous advantages for competition and survival, including the ability to form stable partnerships and shield against toxics and desiccation (Tu et al. 2020; Rummel et al. 2021). Biofilm formation begins with microbial adhesion to the substratum, progressing from weak to stronger interactions and ending with irreversible bonds. As these adherent cells continue to proliferate, they form increasingly complex and mature biofilm communities Besides, external biofilm layers can enhance cell dispersal, promoting colonization of new environments (Renner and Weibel 2011; He et al. 2022). The high surface area of MPs favors the microbial attachment and proliferation onto their surface (Zettler et al. 2013; Frère et al. 2018). Consequently, various microbial species coexist in a reduced space, strengthening their interactions and symbiotic relationships, through *quorum sensing* mechanisms. The term “plastisphere” was coined to describe the distinctive niche of microorganisms present in plastic biofilms (Zettler et al. 2013). Plastic properties, influenced by surface roughness and charges, buoyancy, and polymer composition, play a crucial role in early biofilm formation stages (Sooriyakumar et al. 2022). Several studies showed that microbial diversity in biofilm varies significantly depending on polymers composition (Frère et al. 2018; Miao et al. 2021; Wang et al. 2021). Other factors affecting biofilm establishment and succession include surface availability, morphology of the material, and environmental conditions (Dussud et al. 2018; Xu et al. 2019; Jachimowicz et al. 2022).

Biofilm formation on MPs in natural environments possesses several consequences, that are described as follows.

### Vertical transport and physical transformation of MPs

Biofilm formation on MPs surface, increases their stickiness and density, primarily through the production of extracellular polymeric substances, enhancing the formation of aggregates and creating higher-density complexes (Sooriyakumar et al. 2022). In aquatic environments, this alters MPs buoyancy, causing vertical transport as complexes sink to deeper layers of the water column, and concentrates them in the lower levels. This phenomenon may explain the observed higher concentrations of MPs in sediment compared to water (Lobelle and Cunliffe 2011; Srihari et al. 2023; Ghanadi et al. 2024; Sbarberi et al. 2024). Similar accumulation occurs in soil, where MPs aggregate and accumulate in lower layers. Water infiltration from rainfall and irrigation, along with the presence of plant roots, have been highlighted as indirect factors influencing this phenomenon (Li et al. 2021).

Biofilms formation also alters their surface roughness, hydrophobicity, and specific surface area (Dussud et al. 2018; Tu et al. 2020). The development of biofilms may increase the specific surface area available, thereby enhancing both biodegradation and weathering process (Rummel et al. 2017). As for hydrophobicity, it usually decreases with biofilm formation, although other factors may also contribute. Other factors known to influence the hydrophobicity of MPs include environmental conditions such as salinity and UV exposure, and changes in chemical composition and surface charge induced by the biofilm (Tu et al. 2020).

### Biodegradation

Microbial degradation can affect microplastics depending on the polymer type and the colonizing microorganisms on their surface. Plastics biodegradation involves several steps: biofragmentation and biodeterioration, which consist of breaking down of materials by microbial action by the action of extracellular enzymes able to depolymerize plastic polymers, resulting in the release of lower molecular compounds; followed by assimilation and mineralization, processes in which these compounds are ultimately converted into inorganic substances. Regardless of polymer type, the biodegradation of MPs can also be influenced by environmental variables, including temperature, moisture, UV radiation, pH and oxygen availability (Amobonye et al. 2021; Jachimowicz et al. 2022). For instance, the combination of heat and moisture is known to increase MPs solubility and enhance bond scission. This leads to the formation of new functional groups, thereby increasing the availability of sites for microorganisms to act on, consequently accelerating their biodegradation rate (Chamas et al. 2020; Amobonye et al. 2021). UV radiation, strongly influenced by geographical location and radiation angle, may act as photo-oxidative inducer of polymers, generating oxygen free radicals within their structure. This leads to a decrease in their integrity and thus promotes biodegradation. UV radiation is also known to reduce polymers hydrophobicity, favoring microbial colonization (Montazer et al. 2018; Taghavi et al. 2021). In marine systems, salinity affects photo-degradation by forming salt crystals on MPs surfaces, reducing light absorption, and consequently minimizing its impact on degradation (He et al. 2023b). For its part, oxygen promotes photo-oxidation, favoring degradation mechanisms of plastics (Cai et al. 2018).

Microorganisms from diverse groups and origins have been identified as biodegraders of various plastic polymers, including petroleum-based ones. These microorganisms usually contain enzymes with depolymerizing capacity, thereby being able to actively shorten polymerized chains and degrade polymeric materials. Notable examples include degraders of PE, such as bacterial species from the genera *Lysinibacillus* and *Brevibacillus* and the fungus *Penicillium simplicissimum* ( Yamada-Onodera et al. 2001; Hadad et al. 2005; Jeon et al. 2021), PET, such as the bacterium *Thermobifida fusca* and species from the genera *Acidovorax* and *Ideonella* (Roth et al. 2014; Yoshida et al. 2016; Jachimowicz et al. 2022) and PP, such as bacteria from the genera *Lysinibacillus*, *Serratia* and *Enterobacter* and the fungus *Phanerochaete chrysoporium* (Shimpi et al. 2018; Jeon et al. 2021; Wróbel et al. 2023), all of which are widely used and prevalent as environmental pollutants (Table 1). Regarding bioplastics, despite their limited presence in natural environments, extensive research has identified microorganisms capable of biodegrading them. Examples include degraders of polyhydroxyalkanoates (PHAs), such as bacteria from the genera *Pseudomonas, Bacillus, Ruegeria*, and *Vibrio* (Boyandin et al. 2012; Marín et al. 2023), polybutylene adipate terephthalate (PBAT), such as the bacterial genera *Marimonas* and *Thermobifida* (Delacuvellerie et al. 2021; Jia et al. 2023), PLA, such as bacteria from the genera *Actinomadura*, *Streptomyces*, *Pseudomonas*, and a consortium bacteria from plastic waste (Sriyapai et al. 2018; Noor et al. 2020; Mistry et al. 2022) and polybutylene succinate-co-adipate (PBSA), such as members from the bacterial genera *Actinomadura* and *Laceyella* and from the fungus *Aspergillus* (Sriyapai et al. 2018; Chien et al. 2022).

### Dispersion of microorganisms and gene transfer

Because MPs are small and lightweight, they can act as vectors for microorganisms, facilitating the long-distance transport, especially in aquatic environments, which are susceptible to wind flows and water currents (Sun et al. 2021; Sooriyakumar et al. 2022). This introduces microorganisms attached to the MPs surface into new potential habitats, posing environmental and public health risks if biofilms harbor human and animal pathogens and/or invasive species (Frère et al. 2018; Delacuvellerie et al. 2022; Sooriyakumar et al. 2022; Moyal et al. 2023). Additionally, MPs can serve as active hotspots for horizontal gene transfer (HGT), i.e. transmission of genetic material between bacterial cells (Sun et al. 2021; Wang et al. 2023). While HGT can occur naturally, MPs biofilms provide favorable conditions for genetic information transmission, with a high density of bacterial cells in a confined space and an active metabolic activity (Wang et al. 2023). This can significantly increase the transfer of resistance genes, including those for heavy metals and antibiotics (Sun et al. 2021; Laganà et al. 2019; Moyal et al. 2023). Particularly concerning is antibiotic resistance, attributed to the release of antibiotics into the environment and promotes the development of resistance in bacteria colonizing MPs (Atugoda et al. 2020).

### Sorption and transport of pollutants

The presence of biofilms on MPs can significantly influence the sorption of pollutants, such as antibiotics, hormones, pesticides, and heavy metals, consequently affecting their mobility (Atugoda et al. 2020; Cui et al. 2023). This is attributed to extracellular polymeric substances, which provide additional surface area and functional groups, enhancing MPs’ sorption capacity. Moreover, the biofilm itself, can function as a sorbent and may alter the surface properties of the MPs, influencing its interactions with pollutants. The effects vary depending on biofilm composition, MP type, and pollutant nature, with potential variations in sorption enhancement or mitigation (Li et al. 2018; Cui et al. 2023). External factors, such as ionic strength and the presence of organic matter, also play a role in pollutants sorption (Zuo et al. 2019; Atugoda et al. 2020). Another issue arises when organisms ingest MPs with sorbed pollutants, leading to the accumulation of these contaminants throughout the food chain, phenomenon known as biomagnification. (Atugoda et al. 2020; Cui et al. 2023). Several studies have shown a significant increase of environmental pollutants in organisms such as great shearwaters (*Puffinus gravis*), zebrafish (*Danio rerio*), Japanese medaka (*Oryzias latipes*) and rainbow fish (*Melanotaenia fluviatilis*) in correlation with the ingestion of MPs (Ryan et al. 1988; Rochman et al. 2013; Wardrop et al. 2016; Batel et al. 2016).

### Assessment of the interactions between microplastics and biofilms

Studying the crucial relationships between biofilms on MPs is of outmost importance, with ongoing research yielding varied results depending on environmental conditions, specific attributes of MPs and biofilms, and sampling methods. Numerous techniques are employed for assessing the interactions between MPs and biofilms. In the following lines, a brief outline is provided containing the most common biofilm-related techniques used nowadays, followed by a summary of their applications in scientific publications focused on MPs in natural environments (Table 2).

**Table 2:** Examples of the main techniques employed for the study of the relationships between microplastics and biofilms

Environmental sample	Techniques	Main findings	Reference
Seawater	Metagenomic analysis, detection of pathogens and metaproteomics	Presence of highly active microbial communities within the plastisphere Polymer composition did not affect microbial community structure but influenced expressed functions Hydrocarbon degraders and pathogenic bacteria detected	Delacuvellerie et al. (2022)
Seawater	EM, FC, bacterial production, enzymatic activity, metagenomic analysis	Biofilm growth occurred independently of plastic type during the initial colonization phase Signs of biodegradation observed after 1 month, particularly for bio-based and some fossil-based materials Continuous biofilm growth and increased enzymatic activities found on specific plastics OTUs involved in polymer biodegradation identified, suggesting new insights into plastic biodegradability in seawater	Odobel et al. (2021)
Seawater	Biofilm quantification, SEM, CLSM, FTIR, metagenomic analysis	Biofilm thickness increased over time but decreased with depth Biofilm formation decreased hydrophobicity and increased abundances of hydrophilic groups Dominant bacterial families varying during phases of biofilm formation	Tu et al. (2020)
Seawater	EM, enzymatic activity, total carbohydrates, organic carbon and nitrogen	Environmental conditions impacted biofilms characteristics Summer conditions favored photoautotrophic organisms, leading to decreased prokaryotic abundance Temperature variation in winter delayed enzymatic activity increase, suggesting a time lag in organic matter recycling Correlations indicate tighter links between prokaryotic abundance and environmental variables under temperature alteration Temperature increase and light limitation due to plastic sinking may alter biofilm communities, enhancing the role of prokaryotic organisms	Misic and Covazzi Harriague (2019)
Freshwater	Biofilm quantification, SEM, metagenomic analysis, detection of degraders and pathogens	All MP types exhibited signs of degradation during exposure Community structure and composition differed from those of surrounding water, being the dominant phyla Proteobacteria, Chloroflexi, Verrucomicrobiota, Actinobacteriota, and Firmicutes Plastic-degrading and pathogenic bacteria detected MP type and exposure time significantly influenced bacterial community structure and functional properties Findings highlight the potential ecological impact of MPs on freshwater ecosystems	Song et al. (2024)
Freshwater	Metagenomic analysis	Particle-associated microorganisms in water were the primary source of plastisphere prokaryotes, while free-living microorganisms in water contributed more to plastisphere eukaryotes Prokaryotes showed stronger response to MP than eukaryotes Colonization time determined composition of communities Findings suggest that the assembly of microbial communities in the plastisphere depended more on specific microbial sub-communities and colonization time than polymer types and colonization site	Zhang et al. (2024)
Freshwater	Metagenomic analysis, FISH-CLSM, SEM	Differences in microbial community composition observed between biofilms and corresponding planktonic populations, suggesting selective adhesion on MPs A core microbiome comprising known biofilm formers from freshwater ecosystems was identified, such as *Sphingorhabdus, Sphingomonas, Rhodobacter, Aquabacterium,* and *Acidovorax* genera Species composition of plastisphere did not differ between polymers but a correlation with exposure time was observed Generalist planktonic taxa were found on MPs with lower degradation levels and biodiversity increased with degradation	Di Pippo et al. (2020)
Soil	Metagenomic analysis, detection of pathogens, ARG and virulence factors	PBAT/PLA-MPs had a greater diversity and abundance of ARGs and VFs compared to soil and PE-MPs Actinobacteria predominantly hosted tetracycline and glycopeptide resistance genes in soil and PE-MP plastisphere, while Proteobacteria hosted multidrug resistance genes in PBAT/PLA-MP plastisphere Human pathogens identified The transfer potential of ARGs was higher in PE-MP plastisphere than in PBAT/PLA Findings highlight potential environmental risks associated with MPs in farmland	Li et al. (2024)
Soil	Biofilm quantification, metagenomic analysis	Amount of biofilm on MPs increased with time and MP concentration Polysaccharide in EPS differed among plastics but protein content did not change Dominant bacterial species varied by MP type and concentration, with *Methylophaga*, *Saccharimonadales*, and *Sphingomonas* being prominent Functional prediction suggested involvement in carbon cycling and climate regulation, with LDPE exerting a greater impact on the carbon cycle The interaction between MPs and microorganisms in greenhouse agriculture may affect plant development, soil nutrients, and pose ecological hazards due to MPs biofilms	Chen et al. (2022)
Soil	SEM, metagenomic analysis	Bacterial communities on microplastics differed from those in surrounding soil, plant litter, and macroplastics MPs acted as a “special microbial accumulator” in farmland soil, enriching taxa capable of PE degradation, such as Actinobacteria, Bacteroidetes, and Proteobacteria Co-occurrence network analysis showed complex microbial interactions on MPs, with Acidobacteria, Chloroflexi, Gemmatimonadetes, and Bacteroidetes identified as keystone species	Zhang et al. (2019)

^*^ARG antibiotic resistance genes; *CLSM* confocal laser scanning microscopy; *EM* epifluorescence microscopy; *EPS* extracellular polymeric substances; *FC* flow cytometry; *FTIR* Fourier transform infrared spectroscopy; *LDPE* low-density polyethylene; *OTU* operational taxonomic unit; *SEM* scanning electron microscopy; *VF* virulence factors

### Microbial biofilm composition and dynamics: microbiological characterization

Knowing the genera and species of the microorganisms that make up the biofilm, from the initial stages of colonization to its full maturation, and examining how this process is influenced by the polymeric material, environmental conditions, and even geographic location, plays a crucial role in predicting potential consequences for the ecosystems. Metagenomic analysis, using high-throughput sequencing techniques, is widely employed. Also referred to as new-generation sequencing, it produces large amounts of genetic information through several possible sequencing methods, such as Illumina, HiSeq, and many others. Their use has proved to be a valuable tool for microbial identification and the study of microbial diversity within established communities in aquatic and terrestrial environments (Table 2). This approach also allows the identification of pathogens and study of the presence and/or abundance of genes responsible for antibiotic and heavy metals resistance, and virulence factors within the MPs biofilms. This research offers insights into the dissemination of pathogens and antimicrobial resistance, particularly relevant for public health (Nath et al. 2023; Moyal et al. 2023; Li et al. 2024). Furthermore, since the plastisphere can harbor microorganisms involved in polymer degradation or organic pollutant absorption and/or degradation, the search of these specific functions and the quantification of the expression of related genes can provide interesting information for bioremediation purposes. Metaproteomic and molecular techniques, such as qPCR are useful tools. In this line, Oberbeckmann et al. (2021) employed a metagenomic/proteomic approach to investigate the biodegradation potential and pathogen vectoring properties of PE and PS in a marine environment. They found no evidence of biodegradation or pathogen transmission associated with these plastics. Similarly, Delacuvellerie et al. (2022) detected hydrocarbon degraders and pathogenic species within plastics biofilms but demonstrated their limited activity by the absence of proteins involved in polymer degradation or pathogenicity.

For the characterization and identification of the microbial morphology comprising MPs biofilms, alternatives commonly used entail microscopic observation-based techniques. The use of epifluorescence microscopy, scanning electron microscopy (SEM), and confocal laser scanning microscopy permits the morphological monitoring of the biofilm formation on the MPs surface and its characterization (Tu et al. 2020). Particurlarly, confocal laser scanning microscopy can be coupled with fluorescence in situ hybridization for additional information, such as community composition. Di Pippo et al. (2020) used this approach to study the biodiversity and structure of the communities present on MPs-associated biofilms in a lentic ecosystem, unveiling a patchy colonization pattern of the MPs with a complex biofilm structure; while this technique allowed Vaksmaa et al. (2021) to report that the composition of microbial communities of biofilms on MPs in seawater, was influenced by the type of polymer. Flow cytometry is another technique capable of sorting, counting, and analyzing microorganisms, based on light scattering and fluorescence. It is a fast-counting method that provides viable results (Table 2).

Studying heterotrophic bacterial production in biofilms is crucial for understanding their role in plastic degradation and associated environmental implications. This involves measuring ^3^H-leucine incorporation into bacterial proteins and determining radioactivity (Odobel et al. 2021). As a complement to the expression of genes related to the enzymatic activity, the enzymatic activity of the bacteria of the biofilms can also be determined directly. Examples of enzymes whose activity may be determined include proteases, esterases, glucosidases, lipases and cellulases, among others. The study of these enzymes provides interesting information regarding the establishment of biofilms on MPs and their degradation. In general, relevant enzymes function through two main mechanisms: surface modification and degradation. Surface modification involves hydrolases such as lipases and carboxylesterases, which can alter the surface properties of MPs and therefore enhance biofilm establishment. Degradation mechanisms involve enzymes capable of breaking down the polymer structure such as oxidases and peroxidases (Debroy et al. 2021).

### Indirect biofilm quantification

In the reviewed literature, indirect quantification of biofilms on MPs surfaces is commonly used. Crystal violet staining is a commonly used method that provides straightforward results due to its capacity to bind to negatively charged components of bacteria and extracellular polymeric substances (Tu et al. 2020; Chen et al. 2022). Another frequently used method based on spectrophotometric measurements is the determination of biofilm polysaccharide and protein content, which are positively correlated with biofilms (Misic and Covazzi Harriague 2019; Ganesan et al. 2022; Chen et al. 2022).

### Physicochemical characterization

Given the impact of biofilms on the physicochemical properties of MPs, their investigation is a prevalent approach in the literature. Microscopy techniques yield insights into the effects of biofilm presence on MPs, including changes in texture, color, shape, and potential signs of degradation associated with biodegradation. Common approaches involve the use of stereomicroscopes, and atomic force microscopes, while SEM can generate high-definition images of the MP-biofilm surface. Additionally, coupling these methods with spectroscopic ones (e. g., SEM coupled with energy-dispersive X-ray spectroscopy) can offer supplementary information on both surface topography and elemental composition (Kaur et al. 2022; Ganesan et al. 2022).

Changes in chemical composition and functional groups may be addressed by spectroscopic methods, such as Fourier transform infrared spectroscopy and Raman spectroscopy (Tu et al. 2020; Kaur et al. 2022). The assessment of changes in the hydrophobicity of MPs, by measurements of contact angle, and in their surface charges, by measurements of the zeta potential, are other examples of this group of techniques (Ganesan et al. 2022; Wu et al. 2022; He et al. 2023a).

## Current gaps and challenges

The interactions between biofilms and MPs represent a complex and emerging area of research. While there is growing interest in understanding these interactions, several gaps and challenges persist in this field.

The understanding of the complex interactions between biofilms and MPs remains incomplete due to the complexity of the processes involved and the presence of a wide variety of microorganisms simultaneously interacting. Hence, additional research is imperative to elucidate how microorganisms adhere to, colonize, and potentially degrade them. Moreover, biofilm development onto MPs surfaces produces physicochemical alterations on polymeric substrates. Ongoing research in this area can provide further insight into the specific changes and their relationships to the microbial structure and composition of biofilm and how these aspects affect nutrient cycling, microbial communities, and overall ecosystems health. In this context, and as an example, the study of potential alterations of the biochemical balance in aquatic environments as a result of the establishment of biofilms onto MPs could be included (Arias-Andres et al. 2018; Chen et al; 2020a; Zhang et al. 2020). This ongoing research hasthe potential to provide more accurate ecological predictions concerning the future distribution and abundance of biofilm-associated MPs, along with the potential risks associated with the spread of pathogenic microorganisms and pollutants.

Moreover, more studies are needed to provide accurate data on feasible mitigation strategies, including identifying strains with polymer-biodegrading potential and understanding the metabolic pathways involved. This could permit the development of more sustainable approaches to address the current plastic problem… Nevertheless, the vast diversity of metabolic pathways in microorganisms, coupled with the complexity involved in studying them, presents significant challenges to progress. Research in different areas can help address the challenges posed by mitigation strategies. For example, genomic, metagenomic and proteomic research studies may help identify the genes and enzymes responsible for plastic degradation pathways, while bioinformatics and computational biology could assist in analyzing extensive datasets and build predictive models (Zhang et al. 2022). Lastly, biotechnology, particularly through genetic engineering, would allow to optimize strains with enhanced degrading capabilities (Jaiswal et al. 2020; Silva 2021). However, although genetic engineering has been shown to be highly effective in bioremediation for compounds like hydrocarbons and heavy metals, its use in plastic degradation remains at an early stage, presenting a formidable challenge for scientists to innovate new approaches for environmental plastic management (Jaiswal et al 2020).

Within this context, biodegradable polymers are particularly relevant as for their supposedly lower persistence in nature, and their use has been proposed as a promising strategy to alleviate the issue of MPs pollution. Nevertheless, field studies show that their biodegradation is strongly linked to environmental conditions and that it is only complete under optimal conditions. This challenges the initial belief that biodegradable polymers would break down completely in natural environments (Sintim et al. 2020; Marín et al. 2023). Consequently, their persistence in the environment may be greater than initially estimated, raising concerns similar to those observed for conventional polymers. This suggests the need to reassess the efficacy of biodegradable polymers, and highlights the importance of understanding their behavior in environmental conditions for informed decision-making on their use and regulation in the broader context of environmental sustainability.

Despite the well-known issue of MPs pollution, their environmental and toxicological effects are still far from being fully understood (Palmer and Herat 2021). Challenges in identifying ecotoxic signs and correlating them with specific chemical compounds contribute to this lack of information. However, the development and integration of new analytical methods and technologies offer potential solutions, enabling the acquisition of more detailed and feasible results.

Another gap is the frequent inconsistencies between in vitro and *in* vivo experiments. The natural heterogeneity of MPs in terms of shape, size, and type in the environment contrasts with the more homogeneous samples typically used in in vitro experiments, leading to discrepancies. Additionally, in natural settings, MPs undergo changes with aging and continuous exposure to various substances, making it challenging to replicate these dynamic conditions in laboratory (Rummel et al. 2017). Aligned with this, and in the context of studying polymer biodegradation under simulated natural conditions, it is advisable to consider some of the microbial parameters mentioned above. For instance, the effective establishment of biofilms on samples could be checked throughout the experiments using direct methods, such as metagenomics, or indirect methods, like biofilm quantification. Additionally, signs of microbial degradation could be detected through microscopy or Fourier transform infrared spectroscopy analysis. This would facilitate the establishment of a thorough correlation between biofilm formation and polymer degradation. Currently, these aspects are often overlooked in existing standards, which predominantly rely on respirometry activities. This involves continuous or intermittent monitoring of headspace gas concentrations over time using various analytical techniques such as gas chromatography or infrared spectroscopy.

## Innovative solutions to mitigate microplastic pollution

**Fig. 1:**
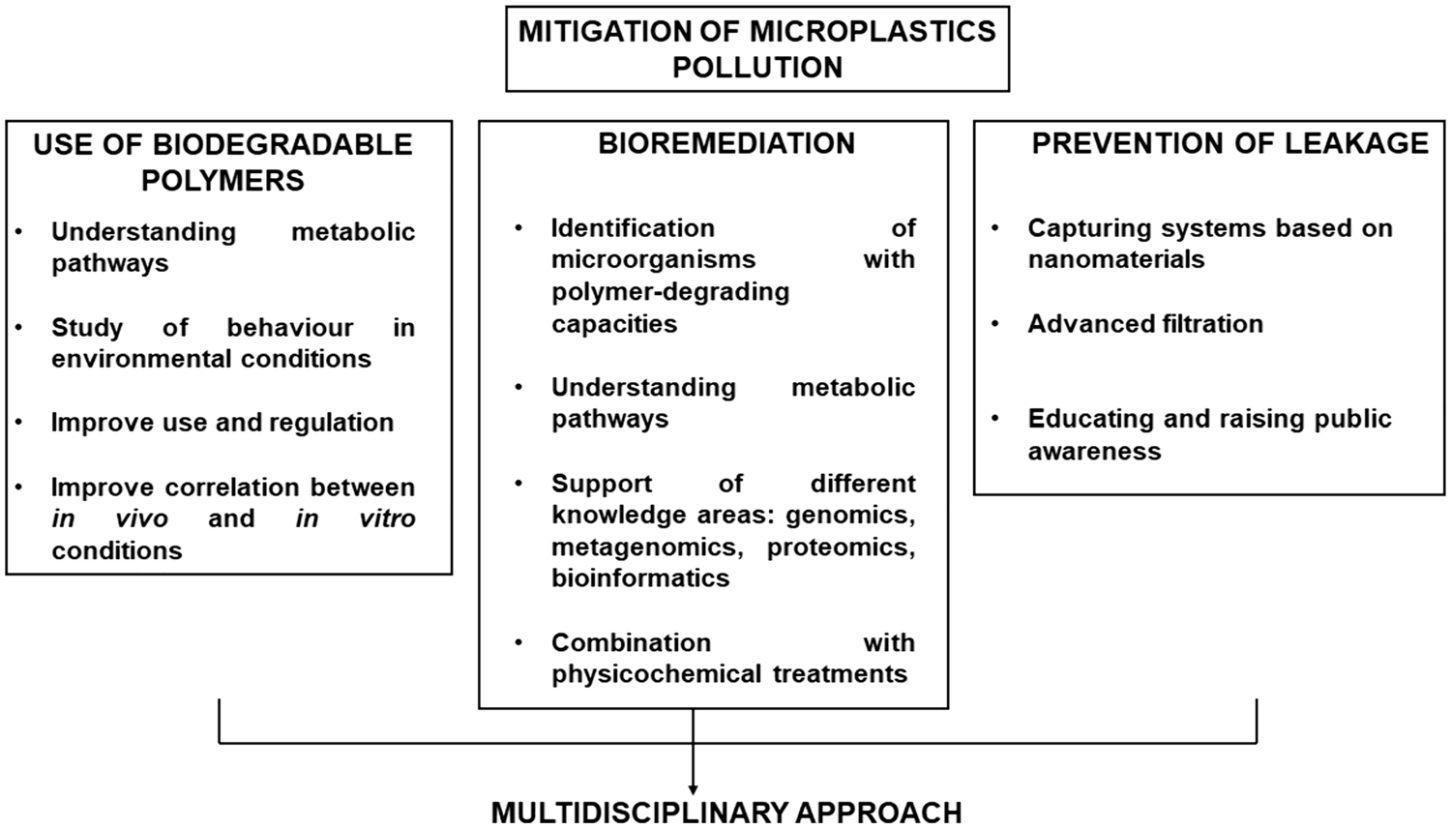
Summary of the main mitigation strategies to fight microplastic pollution

The escalating issue of MPs pollution demands innovative solutions, and bioremediation has emerged as a promising biotechnological tool to address this environmental challenge. Bioremediation aims to leverage the natural biological activities of microorganisms to degrade MPs or transform pollutants into less harmful substances. It is considered more environmentally friendly compared to traditional methods like chemical or mechanical cleanup. This strategy is often applied to contaminated soils and waters (Bansal et al. 2021; Wang et al. 2024a), but its efficiency can be improved following different strategies (Fig. 1).

Combining bioremediation with new-generation strategies such as in silico genome mining integrated with metagenomic datasets, has proven to be both feasible and effective in achieving higher levels of enzymatic activity (Rai et al. 2021; Bansal et al. 2021). In line with this, techniques such as protein and strain engineering and gene-editing tools like CRISPR, can enhance plastic degrading capabilities of microorganisms(Jaiswal et al. 2020; Bansal et al. 2021; Maqsood et al. 2023). Using these techniques, the expression of microbial enzymes relevant to plastics degradation could be enhanced, or alternatively, the genes responsible for encoding them could be inserted into host microorganisms. These genetically modified organisms could be released into contaminated regions to as in the extraction of MPs from the environment. These techniques are innovating as they offer precise control over the introduced genetic modifications, providing efficient and targeted solutions.

Combining biological treatments with physicochemical strategies has also yielded positive results. For instance, Montazer et al. (2018) and Tsiota et al. (2018) evaluated a hybrid approach combining physical and biological methods to degrade plastics. In their studies, PE and LDPE were exposed to UV radiation and then were incubated with specific microorganisms, observing an enhanced degradation efficiency. Furthermore, given the diversity of polymer types, employing multiple microorganisms consortia could enhance the biodegradation of various polymers simultaneously. An example of the use of consortia is the work published by Skariyachan et al. (2021), who observed higher degradation levels of low-density polyethylene (LDPE) and PP with the use of a novel bacterial consortium including *Enterobacter* and *Pseudomonas* strains with potential to be used in biodigesters for industrial applications (Skariyachan et al. 2021). These authors reported that greater adaptability and co-metabolism of the isolates as probable reasons for this enhanced degradation. Also in this line, Pattanasuttichonlakul et al. (2018) combined UV irradiation and a microbial consortium from dairy wastewater to accelerate the biodegradation of PLA, reporting a synergistic effect. Thus, an interdisciplinary approach complemented by sustainable policies is considered the most effective way forward, offering efficient management tools for mitigating microplastic pollution in the environment. Introducing biodegradable polymers as an alternative to traditional plastics is also a key strategy, despite the drawbacks related to their degrading behavior under natural conditions. In this sense, researchers are continously developing and refining biodegradable materials to broaden the conditions under which their biodegradation is possible.

Moreover, nanotechnology offers potential solutions for MPs removal. Nanomaterials with specific properties can be designed to attract, trap, or degrade MPs in water bodies (Scaria et al. 2023; Rajput et al. 2024). However, the potential environmental impacts and unintended consequences of introducing nanoparticles into ecosystems must be carefully considered.

From a prevention perspective, the use of advanced filtration technologies is crucial to capturing MPs before they enter natural ecosystems. Innovations in filtration systems, such as the development of specialized filters and membranes, can help prevent the release of MPs from various sources, mainly WWTPs. The use of flocculation and coagulation technology has been shown to be effective for this purpose (Hidayaturrahman and Lee 2019; Chen et al. 2020b).

Finally, raising public awareness and promoting education about the consequences of MPs pollution are essential components of any comprehensive solution. Encouraging responsible plastic use, promoting recycling, and fostering a broader understanding of the environmental impact of plastics can help reduce the overall influx of MPs into natural environments (Willis and Fytianos 2022).

These innovative solutions are part of a multifaceted approach to addressing the complex and pervasive problem of MPs pollution. While progress is being made, continued research, collaboration, and the adoption of sustainable practices are crucial to effectively combat MPs in natural ecosystems.

In conclusion, MPs and biofilms formed on their surface in natural environments establish complex interactions which alter MPs physicochemical traits and give rise to relevant issues. Among these, bioaccumulation and the dissemination of pathogens, invasive species, pollutants, and resistance genes pose serious threats to both human and ecosystems health. Understanding these interactions is crucial for the development of mitigation strategies, which requires the integration of scientific research, technological advances, and public engagement. Through a multidisciplinary approach, the current gaps and challenges can be gradually effectively addressed.

## Data Availability

No datasets were generated or analysed during the current study.
